# Identification and Analysis of Genes Underlying Bone Mineral Density by Integrating Microarray Data of Osteoporosis

**DOI:** 10.3389/fcell.2020.00798

**Published:** 2020-08-27

**Authors:** Haihong Zhang, Jinghui Feng, Zhiguo Lin, Shuya Wang, Yan Wang, Siming Dai, Weisi Kong, Yanli Wang, Zhiyi Zhang

**Affiliations:** ^1^Department of Rheumatology and Immunology, The First Affiliated Hospital of Harbin Medical University, Harbin, China; ^2^Department of Gerontology, The First Affiliated Hospital of Harbin Medical University, Harbin, China

**Keywords:** bone mineral density, osteoporosis, microarray, co-expression, enrichment analysis

## Abstract

Osteoporosis is a kind of brittle bone disease, which is characterized by a reduction in bone mineral density (BMD). In recent years, a number of genes and pathophysiological mechanisms have been identified for osteoporosis. However, the genes associated with BMD remain to be explored. Toward this end, we integrated multiple osteoporosis microarray datasets to identify and systematically characterize BMD-related genes. By integrating the differentially expressed genes from three osteoporosis microarray datasets, 152 genes show differentially expressed between high and low BMD osteoporosis samples in at least two of the three datasets. Among them, 88 were up-regulated in high BMD samples and 64 were up-regulated in low BMD samples. The expression of ZFP36, JUNB and TMEM8A were increased at high BMD samples in all three datasets. Hub genes were further identified by co-expression network analysis. Functional enrichment analysis showed that the gene up-regulated in high BMD were enriched in immune-related functions, suggesting that the immune system plays an important role in osteoporosis. Our study explored BMD-related genes based on the integration of osteoporosis microarray data, providing guidance to other researchers from a new perspective.

## Introduction

Osteoporosis, a common systemic bone disease, can lead to weak bones and increase the risk of fractures (Ensrud and Crandall, 2017; Wilson et al., 2017; Khosla et al., 2018). A third of women and a fifth of men over the age of 50 have broken bones due to osteoporosis (Brown, 2017). Osteoporosis is characterized by low bone mass, microstructure degeneration and reduced bone strength. Patients often have a reduction in bone mineral density (BMD) (Coughlan and Dockery, 2014; Black and Rosen, 2016). Bone homeostasis depends on osteoclast absorption and osteoblast formation. The imbalance of this tightly coupled process can lead to the development of osteoporosis (Chen et al., 2018b). This process involves changes in a variety of signaling pathways, including MAPK signaling pathway, NF-κB pathway, Notch signaling pathway, etc. (Chen et al., 2018a, 2019; Lee and Long, 2018; Wang et al., 2019). Notch signaling pathway regulates the differentiation and function of osteoblasts and osteoclasts and participates in the process of bone reconstruction, activation of notch signaling pathway can inhibit glucose metabolism and osteoblast differentiation of bone marrow mesenchymal progenitor cells (Lee and Long, 2018).

In recent years, some genes and pathophysiological mechanisms have been identified by microarray analysis for patients with osteoporosis (Liu et al., 2005; Lei et al., 2009; Li et al., 2016). The study based on peripheral blood monocyte cells (PBMCs) microarray data of osteoporosis patients revealed the pathophysiological mechanism of osteoporosis, which is characterized by increased recruitment of monocytes into bone and then differentiating into osteoclasts (Liu et al., 2005). Zhou et al. (2018b, 2019) predicted osteoporosis related transcription factors (TF) and long non-coding RNA (lncRNA) via exon arrays. Another study explored osteoporosis-related pathways based on microarray data (Zhou et al., 2018a). The studies mentioned above were based on a limited set of data, and have certain limitations. In order to obtain more robust results, it is critical to integrate multiple datasets for obtaining new insights.

In this study, we collected three microarray datasets of osteoporosis. Differential expression analysis was conducted to identify differentially expressed genes (DEGs) between high and low BMD samples. Then DEGs were integrated to obtain uniformly expressed BMD-related genes. Analysis of gene co-expression networks revealed key genes of different BMD conditions. And enrichment analysis showed that they were enriched in immune-related functions and biological pathways, suggesting a potential role of the immune system in osteoporosis.

## Materials and Methods

### GEO Datasets

Microarray datasets of high and low BMD osteoporosis samples were downloaded from Gene Expression Omnibus (GEO) database^1^, including GSE2208, GSE56814 and GSE56815. A total of 172 samples were collected from the three datasets, of which 92 were high BMD samples and 80 were low BMD samples (Table 1).

**TABLE 1:** The overview of three GEO datasets.

Dataset	Sample number		Gene number
	High BMD	Low BMD	
GSE2208	10	9	6353
GSE56814	42	31	17321
GSE56815	40	40	13515
Total	92	80	19718

*The total number of gene does not include genes that appear multiple times.*

### Identification of Differentially Expressed Genes (DEGs)

The up-regulated genes in high or low BMD samples of each dataset were identified using the R package named limma with a threshold of |log2FoldChange|>0 and *P* < 0.05. Genes up-regulated in at least two datasets were identified as uniformly expressed BMD-related genes and used for subsequent analysis.

### The Construction of Gene Co-expression Network

We calculated the Pearson Correlation Coefficient (PCC) of integrated up-regulated genes in high or low BMD samples (PCC > 0.4 and *P* < 0.05). For co-expressed gene pairs that appeared in multiple datasets, the averaged PCC was calculated. Then Cytoscape (V.3.8.0) was used for network visualization, and hub genes were identified by cytoHubba plugin (Shannon et al., 2003; Chin et al., 2014).

### Enrichment Analysis

We performed GO and KEGG enrichment analysis of genes included in co-expression network using R package “clusterProfiler” (Yu et al., 2012). The significance threshold is 0.05.

## Results

### Differentially Expressed Genes Between High and Low BMD Osteoporosis Samples

To explore the key genes associated with BMD, we downloaded three osteoporosis microarray datasets from NCBI GEO database. In total, 19718 genes and 172 samples were involved. The number of high and low BMD samples in each dataset was comparable (Table 1).

**FIGURE 1:**
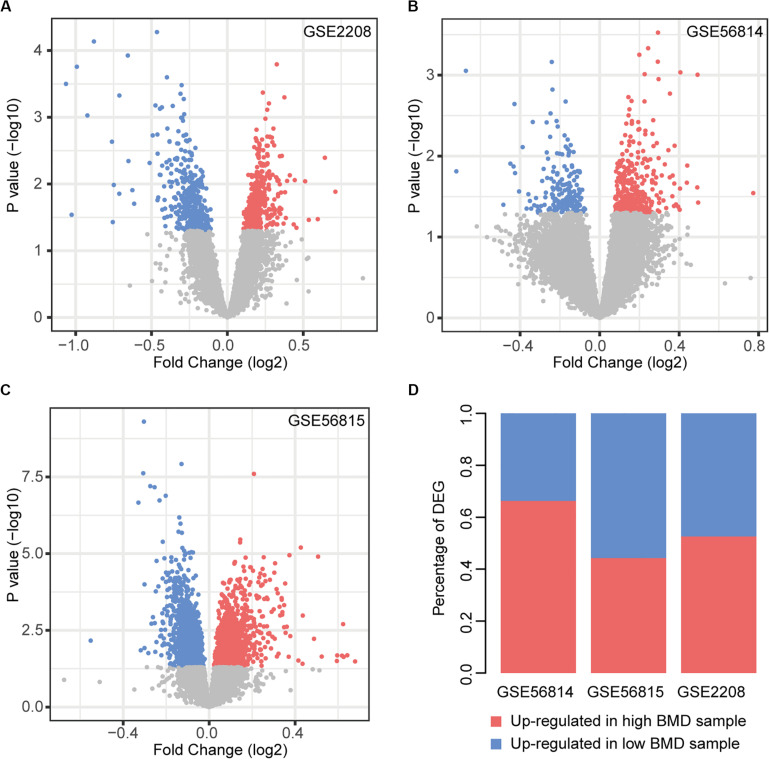
Differential expression analysis. **(A–C)** Volcano plot of DEGs in each dataset, red nodes represent upregulation in high BMD samples and blue nodes represent upregulation in low BMD samples. **(D)** Statistics of two types of DEGs.

We performed the analysis of differentially expressed genes between the two types of samples (Figure 1). There were 12.59% (GSE2208), 3.30% (GSE56814), and 18.49% (GSE56815) genes identified as differentially expressed, respectively. In GSE56814, compared to the low BMD samples, there were more genes up-regulated in high BMD samples, while GSE56814 had more genes up-regulated in low BMD samples. In GSE2208, the two types of genes were almost the same in number (Figure 1D).

### Integration of BMD-Related Genes

**FIGURE 2:**
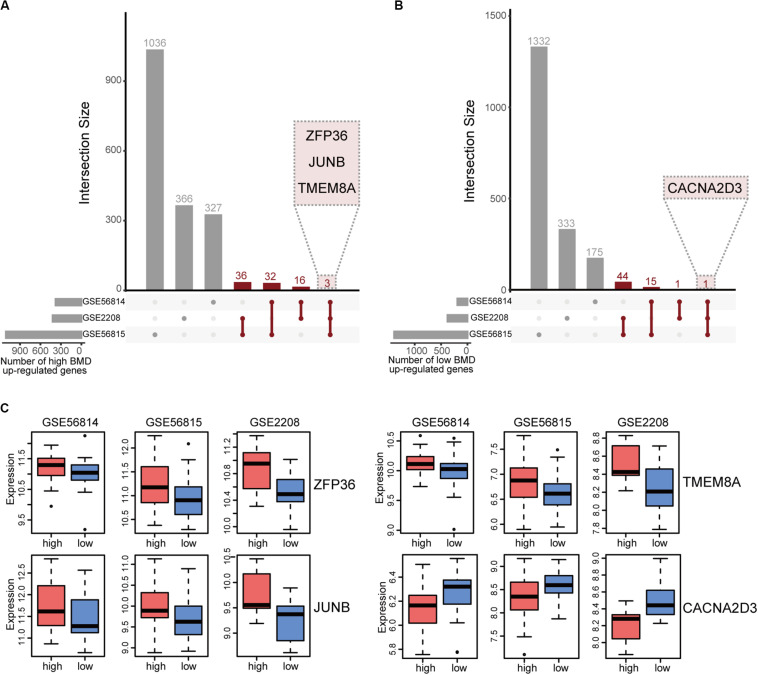
Integration of BMD-related DEGs. **(A)** UpSetR plot of genes up-regulated in high BMD samples, the number of genes present in at least two datasets is marked (red). **(B)** UpSetR plot of genes up-regulated in low BMD samples, the number of genes present in at least two datasets is marked (red). **(C)** Boxplot of genes that expressed consistently across all three datasets.

We integrated the DEGs of three datasets to obtain robust different BMD-related genes. Based on the criterion that the same gene was up-regulated in at least two datasets, we identified 88 and 64 uniformly expressed genes with high and low BMD by UpSetR, respectively (Figures 2A,B and Supplementary Tables 1, 2; Conway et al., 2017). In all datasets, there were three genes, ZFP36, JUNB and TMEM8A shown up-regulated in high BMD (Figure 2C and Table 2). JUNB is a member of c-Jun protein family, which interacts with c-Fos protein family to form transcription factor AP-1. AP-1 can activate osteoclast specific genes (Wagner and Eferl, 2005; Asagiri and Takayanagi, 2007; Hamamura et al., 2015). Only one gene, CACNA2D3, had elevated expression with low BMD in all datasets (Figure 2C and Table 2).

**TABLE 2:** Genes expressed consistently across all three sets of data.

Gene	GSE2208		GSE56814		GSE56815	
	
	log_2_FC	P	log_2_FC	P	log_2_FC	P
ZFP36	0.355	0.024	0.216	0.042	0.274	0.004
JUNB	0.512	0.009	0.249	0.049	0.305	0.006
TMEM8A	0.279	0.024	0.134	0.021	0.211	0.013
CACNA2D3	−0.256	0.015	−0.111	0.015	−0.268	0.001

### Co-expression Network and Functional Analysis of BMD-Related Genes

**FIGURE 3:**
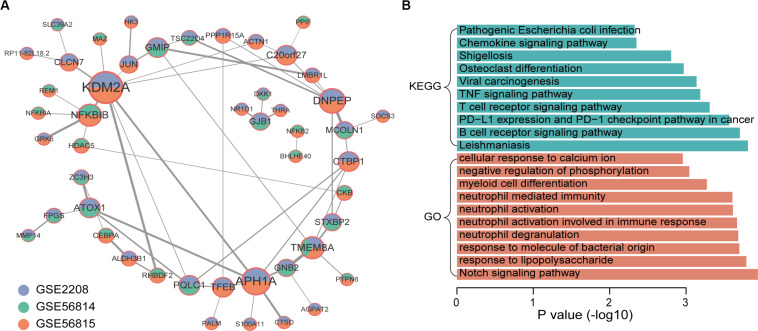
Co-expression network and function enrichment analysis of genes that are consistently expressed in high BMD samples. **(A)** The gene co-expression network. Node size represents how many other genes interact with it. Node color indicates in which data sets it is up-regulated in high BMD samples. The darker the edge color, the greater the correlation coefficient. **(B)** GO and KEGG results of genes in network.

Through the similarity of gene expression, the possible interaction between gene products can be analyzed, so as to understand the interaction between genes and find out the core genes. Therefore, we performed a co-expression analysis of integrated up-regulated genes in high and low BMD samples, respectively. We obtained 58 co-expressed gene pairs of high BMD. The top5 hub genes were identified by cytoHubba, a plug-in of Cytoscape, including KDM2A, APH1A, DNPEP, NFKBIB, and TMEM8A. KDM2A was co-expressed with the largest number of genes (Figure 3A). KDM2A can regulate Mesenchymal stem cells (MSCs) osteo/dentinogenic differentiation and cell proliferation (Dong et al., 2013; Gao et al., 2013). Co-expression network of genes that were up-regulated in high BMD samples contained 48 gene nodes. We did functional enrichment analysis on them. In addition to osteoporosis-related functions and pathways, Notch signaling pathway and osteoclast differentiation, enrichment analysis results showed that they were mainly enriched in immune-related functions and pathways (Figure 3B; Boyle et al., 2003; Lee and Long, 2018). Studies have shown that the immune system plays an important role in osteoporosis (Faienza et al., 2013). For example, postmenopausal osteoporosis patients had higher T-cell activity and increased TNFα and RANKL production, which can promote osteoclast differentiation (D’Amelio et al., 2008; Mirza et al., 2010; Kim et al., 2012). In addition, other studies have shown that B lymphocytes were closely related to bone metabolism (Takayanagi et al., 2005; Boyce and Xing, 2008; Breuil et al., 2010).

**FIGURE 4:**
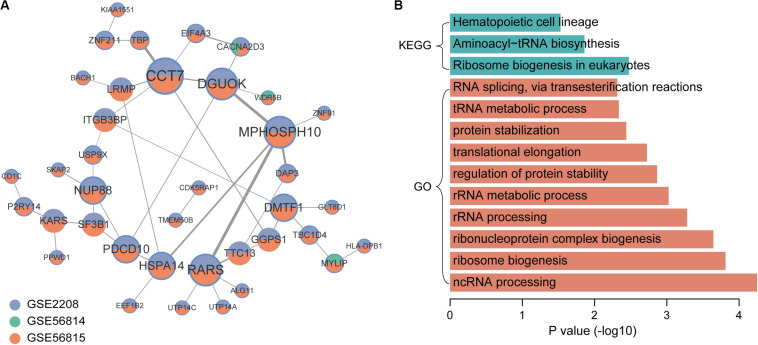
Co-expression network and function enrichment analysis of genes that are consistently expressed in low BMD samples. **(A)** The gene co-expression network. Node size represents how many other genes interact with it. Node color indicates in which datasets it is up-regulated in low BMD samples. The darker the edge color, the greater the correlation coefficient. **(B)** GO and KEGG results of genes in network.

In the co-expression network of genes that were up-regulated in low BMD samples, there were 44 gene pairs, involving 38 genes (Figure 4A). The top5 hub genes were CCT7, DGUOK, MPHOSPH10, RARS, and DMTF1. The results of enrichment analysis showed that low BMD-related genes were mainly enriched in basic biological processes and pathways, including ribosome biogenesis, rRNA processing and translation elongation (Figure 4B).

## Discussion

In this study, we identified and analyzed BMD-related genes through three osteoporosis microarray datasets. Differential expression analysis identified DEGs between high and low BMD in each dataset. By integration, we screened out 152 uniformly differentially expressed genes. Gene co-expression network analysis further identified key top 5 hub genes. Co-expression genes that had elevated expression in high BMD were enriched in functions of immune systems, suggesting its important potential role in osteoporosis. In addition, they were also enriched in osteoclast differentiation pathway, indicating that high BMD is developing toward low BMD.

In conclusion, we used bioinformatic methods to systematically characterize genes underlying BMD levels based on osteoporosis microarray data. We analyzed osteoporosis from a new perspective and provided new guidance for its diagnosis and treatment.

## Data Availability Statement

All datasets presented in this study are included in the article/Supplementary Material.

## Author Contributions

ZZ designed the study. HZ, JF, ZL, SW, and YW analyzed the data. SD, WK, and YLW wrote the manuscript. All authors read and approved the manuscript.

## Conflict of Interest

The authors declare that the research was conducted in the absence of any commercial or financial relationships that could be construed as a potential conflict of interest.

## Footnotes

1 https://www.ncbi.nlm.nih.gov/geo/

## Supplementary Material

TABLE S1 The uniformly up-regulated DEGs in high BMD samples.

TABLE S2 The uniformly up-regulated DEGs in low BMD samples.
